# Self-regulated learning strategies of Macau English as a foreign language learners: Validity of responses and academic achievements

**DOI:** 10.3389/fpsyg.2022.976330

**Published:** 2022-10-03

**Authors:** Xiaojia Deng, Chuang Wang, Jianzhong Xu

**Affiliations:** ^1^University International College, Macau University of Science and Technology, Taipa, Macao SAR, China; ^2^Faculty of Education, University of Macau, Taipa, Macao SAR, China; ^3^Department of Counseling, Educational Psychology, and Foundations, Mississippi State University, Starkville, MS, United States

**Keywords:** self-regulated learning, academic achievement, validity, English as a foreign language, Macau

## Abstract

Self-regulation is important in enhancing students’ academic performance, yet evidence for the systematic and valid instruments to measure self-regulated learning strategies of college students in an English as a foreign language context is far from robust. This study was situated to develop an evaluation tool to examine the status quo of self-regulated learning strategies employed by college English learners and the associations between the use of these strategies and their academic achievement. A large-scale survey was conducted at a university in Macau to provide evidence of the construct validity of responses to the questionnaire on self-regulated learning strategies. Conceptualized in social cognitive theory, the questionnaire comprised environmental, behavioral and personal self-regulated learning strategies with 48 items weaving into 10 dimensions. Strong evidence for reliability and validity was found. Findings also revealed that students who intrinsically valued and used more self-regulated learning strategies achieved higher academic performance. Students in advanced-level English course reported significantly more frequent use of self-regulated learning strategies than students in medium-level and mixed-level English courses. Our results draw attention to the pedagogical orientation for teachers of English as a foreign/second language in helping students become adaptive learners with self-regulative process.

## Introduction

Since its inception, self-regulation has been accorded great importance as a 21st-century skill (Trilling, 2009). Schools and universities seek to empower students to become self-regulated learners in the recognition that, in this ongoing fast-changing era, it is a core skill that enables them to monitor the quality of their work and adopt strategies to cope with new and demanding tasks (Panadero et al., 2018). Zimmerman (1989) contended that efficient self-regulated learning (SRL) strategies may facilitate achievements in all academic areas when learners are engaged in a cluster of internal processes that promote adjustments to their knowledge, motivation, behavior, and context. In recent years, different investigations in the education field have manifested the significance of students’ SRL strategies for academic success (Kitsantas et al., 2009; Diseth, 2011; Wang et al., 2013; Fauzi and Widjajanti, 2018; Sutarni et al., 2021).

Previous studies on SRL strategies have laid a promising theoretical foundation, but the validity evidence for a systematic instrument to measure college students’ SRL strategies in English as a foreign language (EFL) contexts is far from robust (Chen et al., 2020). It is particularly rare in regions where English teachers normally work with large student cohorts in each class where learners use fewer SRL strategies than their counterparts do in other countries (Ho, 2004; Lee et al., 2009; Li et al., 2018). To bridge this gap, the current study attempts to examine the status quo of SRL strategies employed by college EFL learners and the associations between SRL strategies and their academic achievements. The outcomes could be used to map out how various SRL strategies might impact English-language learning achievements in an Asian context and could be used for cross-cultural comparisons.

## Theoretical underpinnings

### Self-regulated learning

The concept of SRL strategies sprang up in the 1980s under the influence of social cognitive theory (Bandura, 1986). Bandura’s theory acts as an alternative to Vygotsky’s socio-culturalism and Piaget’s constructivism, which depicts learning processes as reciprocal interactions between cognition, behavior, environment, and other contextual or personal factors. Studies on SRL discuss this reciprocity with a triadic analysis of three component processes: self-observation, self-judgment, and self-reaction (Kanfer and Gaelick, 1986; Schunk, 1989; Zimmerman, 1989). Here, SRL connotes a more self-directed learning experience by which a learner attempts to control these triadic factors to attain their learning goals. Zimmerman (2002) developed an SRL model to elucidate how students exert specific learning strategies to acquire knowledge. For Zimmerman, learners who can regulate their learning have a clearer idea of what they are doing and can better transform mental abilities into academic skills through such self-regulatory strategies as monitoring, controlling, adjusting, self-directing, and self-assessing. Grounded in Zimmerman’s construct, Boekaerts and Corno (2005) pointed out that self-regulated learners demonstrate several distinctive characteristics. They can be conceived of as (a) *adaptive* learners with a range of self-regulative processes through which they set goals, manage resources, self-monitor, and seek feedback; (b) *positive* learners who sustain learning interest and show confidence in achieving learning objectives; and (c) *proactive* learners who know how to select the best strategies to suit their abilities, based on their self-motivational belief in their strengths and weakness (Zimmerman, 2002).

For SRL skills to be successfully passed to learners and useful for practice, it is necessary to understand the crucial processes involved in the self-regulation of learning. Zimmerman (2002) proposed that students undergo three main cyclical phases when regulating their learning. In the first phase (*forethought*), learners clarify and share the goals and standards to attain in a certain task. This phase involves students’ perception of the task’s affordances and constraints and their motivation arising from beliefs about learning such as self-efficacy and outcomes expectancy (Ertmer and Newby, 1996; Zimmerman, 2000, 2006). During the second phase (*performance*), learners engage with the task and monitor their learning, usually deploying planned strategies to compare their progress against standards set in the forethought phase and discover causes of learning events. In the third phase (*self-reflection*), learners evaluate their work and generate applicable revisions or adjustments thereto. This includes reflecting on feedback and mentally storing ideas and concepts to use in the task. To conclude, self-regulated learners make deliberate and goal-directed efforts to adjust, adapt, or abandon their learning strategies and identify, retrieve, and seek new information for future learning (Winne, 1995; Zimmerman, 2008).

Students must apply relevant strategies when learning. In the field of language learning, self-regulation is emphasized as a crucial factor that sets the scene for improved language competence (Seker, 2015; Oxford, 2016; Bai, 2018). SRL is important and a pressing need in the EFL context, as learners’ language learning is primarily restricted to classroom settings and lacks sufficient interaction opportunities (Kormos and Csizeìr, 2014). It is worth mentioning, though, that self-regulation skills do not develop spontaneously but must be learned (Winne, 2005). Research studies have identified SRL strategies as teachable skills that students can obtain during learning processes (Oxford, 2011; Andrade and Evans, 2012; Teng and Zhang, 2021). A critical approach is to cater to individual students by integrating explicit instruction on SRL skills into the larger context. Several researchers have highlighted the necessity of teachers’ explicitly training their students in self-regulatory techniques. For example, Tseng et al. (2015) suggested English teachers should teach learning strategies clearly, activate learners’ metacognition, and enhance their self-efficacy. By using systematic instructional approaches in guiding SRL, teachers can help university students improve their capabilities by incorporating goal setting, strategy implementation and monitoring, and problem-solving tactics into the writing process (Lam, 2015). Employing a person-centered data analysis approach, Chen et al. (2020) noted that higher achievers in language learning tend to manipulate various SRL strategies. This implies that language teachers can encourage learners to take advantage of these strategies and enrich underachievers’ awareness of using them. There is a positive and constructive link between SRL strategies and language learners’ academic achievements (Teng et al., 2019).

### Self-regulated learning strategies as related to academic achievement

Calls for promoting learners’ SRL strategies are not accidental, as many prospective, experimental, and even cross-disciplinary studies have paid tremendous attention to their significant associations with academic achievements (Gaskill and Hoy, 2002; Garner, 2010; Zheng, 2016).

In a meta-analysis of SRL by Broadbent and Poon (2015), SRL theories were applied to students’ efforts in a triadic loop dealing with learning performance: monitoring learning performance (*self-observation*), evaluating learning performance (*self-judgment*), and responding to performance outcomes (*self-reaction*). Broadbent and Poon (2015) contended that learning is not viewed as a fixed trait and will be more effective if the participant sets goals to attain academic success. If learners possess improved SRL strategies, they generally have better perceptions of course content and can achieve more favorable outcomes (e.g., Chang, 2005; Moseki and Schulze, 2010).

The most comprehensive set of self-regulatory strategies (i.e., metacognitive self-regulation strategies, cognitive strategies, and environment and resource management) has been widely discussed in the sphere of language learning (DiPerna et al., 2002; Tseng et al., 2006). Published literature has well documented that self-regulated learners manipulate a range of these components as part of their learning process to achieve successful outcomes (e.g., Vianty, 2007; Liu and Feng, 2011; Zhang and Seepho, 2013; Rasooli et al., 2014). Pitenoee et al. (2017) asserted that metacognitive strategies are closely tied to the executive control of cognition, which increases students’ achievement scores. EFL students with good metacognitive strategies can plan, monitor, and evaluate their learning processes, leading to more positive academic outcomes (Yang, 2009). Cognitive strategies include several sub-strategies and are classified into two types of processing strategies—surface cognitive and deep cognitive. Deep cognitive strategies (i.e., elaboration, organization, and critical thinking) improve academic achievement, whereas surface strategies (i.e., repetitive rehearsal and rote memorization) usually have negative associations with academic achievement (Akamatsu et al., 2019). Nevertheless, some researchers note that proficient learners demonstrate the integration of surface and deep cognitive strategies in promoting long-term retention of academic tasks (Wolters, 2004; Bayat and Tarmizi, 2010). Another important dimension of SRL strategies, conceptualized as environment and resource management, comprises regulatory strategies that students apply deliberately to manage other resources besides cognition.

Pintrich et al. (1993) offered more dimensions, including (a) time and study environment management (creating a realistic plan and organizing a congruous setting for learning); (b) effort management (coupling persistence with a concentration on learning tasks); (c) peer learning (learning from a study group or friends); and (d) help-seeking (seeking help from peers or instructors where necessary).

Oxford and colleagues reported a broader range of SRL strategies, with six sub-scales: memory strategies, cognitive strategies, compensation strategies, metacognitive strategies, affective strategies, and social strategies (Oxford and Burry-Stock, 1995; Oxford, 1996). In the field of foreign language learning, Oxford (1996, 2011) blazed a trail for other researchers to follow—she ushered in one of the best-known instrument (i.e., Strategy Inventory for Language Learning) and enhanced Strategic Self-Regulation (S2R) Model. Oxford and her colleagues’ intensive and extensive discussions on the complex nature of applying strategies in language learning have laid profound groundwork for SRL strategies as they draw parallels and cement relatedness among various theories, such as self-regulation, mediated language learning, emotion, and learner autonomy (Oxford, 2015, 2016; Pawlak and Oxford, 2018). Bai and Wang (2020, p. 7) claimed a rather long list with nine types of SRL engagements: “(1) goal setting and planning, (2) record-keeping and monitoring, (3) self-consequences (i.e., students arrange rewards or punishment for themselves), (4) self-evaluation, (5) effort regulation, (6) organization and transformation, (7) rehearsal and memorization, (8) seeking social assistance, and (9) seeking opportunities to practice English.” One interesting finding derived from their study was that seven types of SRL strategies were weighed more often by learners, whereas “seeking opportunities to practice English” and “goal setting and planning” played insignificant roles in directing their choice, effort, cognitive engagement, and academic performance.

Although a plethora of research has discussed the link between SRL strategies and learning achievements, several issues remain. Concerns include theoretically important but empirically uncertain questions regarding the use of SRL strategies among college students in the Macau context, the variables affecting their English language proficiency, and the lack of a valid and reliable instrument to measure their SRL strategies for EFL education. An investigation into these factors would be theoretically intriguing and is urgent for the context of the present study, the Macau Special Administrative Region of China. The fundamental law of Macau education system has placed great emphasis on more quality-concerned English classes and launched the appeal of “nurturing students’ attitude and ability of life-long learning” (Education and Youth Affairs Bureau, 2016). Over the past few years, Education and Youth Affairs Bureau has organized meetings with school English teachers concerning SRL strategies, yet the research of these viable means in assisting students’ language learning is rare. Our study filled this gap by addressing and extending these aspects *via* empirical investigations in the Macau SAR. Such investigations can provide insights to educators in other places similar to Macau.

### Grand challenges in the English as a foreign language context and the call for a valid instrument to measure self-regulated learning

Investigating the challenges and factors that may affect foreign language learning is pivotal for informing theory, practice, and policy. In a highly dynamic and constantly changing educational landscape, researchers and teachers more than ever need to dissect the field’s challenges and move forward to best serve the shifting needs of language learners. Informed by research from recent decades, Hlas (2018) outlined a bevy of current grand challenges in the EFL context, including a deep delve into measuring progress and learner outcomes (e.g., the specific frameworks or tools influencing learner outcomes, the characteristics of effective assessments for scaffolding students’ language production) and the reliability and validity of teaching/guiding tools (e.g., how educators assess the content validity of guiding tools). Hlas (2018) appealed for more investigation of these tools’ validity, echoing calls by Norris and Pfeiffer (2003), Troyan (2012, 2016), and Tigchelaar et al. (2017). Another grand challenge in the domain of EFL conveys an expectation of moving learners toward higher proficiency (Gitomer and Zisk, 2015). Deciphering this challenge entails investigating the various components that influence foreign language learning achievement, such as the relationship between students’ SRL strategy use and language proficiency. Gaining insights into how SRL may promote English language learning achievements can immensely help EFL or English as a second language (ESL) learners tackle their difficulties (Bai and Wang, 2020). As SRL strategies used by learners with various English language proficiency and cultural backgrounds can provide theoretical insights, our survey included mixed-level learners for a comparison.

The language learning environment is also a grand challenge on which research must center (Tsavga, 2011; Copland et al., 2014). According to Kormos and Csizeìr (2014), foreign language learners learn in impotent language environments. Wei and Su (2012) revealed that, despite the growing population of EFL learners in China, only a tiny fraction (7%) use the target language in their daily lives. Even in regions where English has an official/*de facto* function (e.g., Hong Kong and Macau), opportunities to interact with these languages in everyday settings are prodigiously few with large class size (Education and Youth Affairs Bureau, 2001; McKay, 2002; Moody, 2009). By contrast, in the ESL context, learners are immersed in a predominantly English-speaking environment.

The most comprehensive view of these differences is presented in the “circles” model (Kachru, 1985), which classifies the world into three circles. The inner circle refers to places such as the United Kingdom, United States, Australia, or Canada, where English is a native language that ESL learners regularly encounter on the streets, in the media, in public services, and through everyday activities. The outer circle comprises countries whose colonial history means English is widely used and spreading daily, like Malaysia and India. Finally, expanding circle countries are those in which English does not play an official or institutional role and is used in more restricted circumstances as a foreign language. In these countries, attaining higher English proficiency is rarely possible, due to the unconducive language environment; thus, some educators believe SRL is particularly important in these contexts. While research on SRL is increasing, the status quo of SRL strategies used by college students learning English in expanding circle regions like Macau remains an enigma. Little is known about an instrument to measure these EFL learners’ SRL strategies; hence, the current study’s focus.

Along with the language learning environment, the immediate focus on instructional quality is another key challenge for providing continuity of learning, especially during the rapid spread of the coronavirus (O’Keefe et al., 2020). During this challenging time, educators must become aware of specific approaches to improve instruction and foster SRL strategies online and outside the physical classroom, since almost every instructor in 2020 must focus more on classroom-to-remote instruction. Foreign language teaching should adapt to this trend by creating better-blended combinations to improve learning strategies and outcomes. The first step in laying a data-informed basis for language teachers and further cultivating learners’ self-regulation of their individualized needs is having a valid instrument to measure students’ SRL strategies. Given the scant support for examining validity of responses to existing instruments, this paper draws on documentary evidence to discuss the construct validity of a questionnaire measuring college students’ SRL strategies. Validity studies are critical ingredients that can inform, justify, and possibly transform educators’ choices on how to support sustainable learning (Wang and Sun, 2020). In what follows, some research instruments will be elaborated, in correlation with their construct validity for measuring students’ SRL strategies.

### Review of existing instruments

To measure students’ perceptions of their general learning behaviors and cognitive activities, as well as their general motivation and capacity for SRL, several research instruments have been generated. Existing tools such as the Motivated Strategies for Learning Questionnaire (MSLQ; Pintrich et al., 1993), Metacognitive Awareness Inventory (MAI; Schraw and Dennison, 1994), and Learning Strategies questionnaire (LS; Warr and Downing, 2000) have been adopted in a multitude of studies. The psychometric properties of these instruments, however, are regularly questioned in that their predictive value of future learning outcomes is insufficient (Greene et al., 2013; Veenman, 2005). More to the point, these instruments do not cover the full range of SRL activities and are not specifically designed for the field of foreign language learning. Oxford (1990) Strategy Inventory for Language Learning embraced multifold learning strategies regarding memory, cognitive, compensation, metacognitive, affective and social factors, yet did not derive from the self-regulation theory and failed to indicate iterative SRL phases (Wang and Bai, 2017). In the context of EFL writing, Teng and Zhang (2016) developed a multidimensional questionnaire that measures cognitive, metacognitive, social–behavioral, and motivational regulation aspects in Chinese undergraduate students’ writing. Guided by social cognitive concept and SRL framework, Wang and Bai (2017) research instrument presented high internal consistency (0.92) and test-retest reliability (0.79). The external aspect of construct validity of Wang and Bai scale was also high (Chen et al., 2020). However, their instrument was validated based on the data from secondary school students and may not allow researchers to draw conclusions relative to college students due to the possible disparities in the nature and development of SRL between college students and students of other age groups (Schneider, 2008). There is a need to tailor the measurement of SRL strategies to the specific domain (e.g., higher education) and develop a new and valid instrument to particularly deal with EFL learners in Macau context. Therefore, this study is to validate a new instrument designed to measure Macau college students’ use of SRL strategies, i.e., English Self-Regulated Learning Questionnaire (ESRLQ) (see the Appendix).

Wang and Bai (2017) validation study suggested that research on language learner self-regulation could comply with Messick’s (1995) test validity model, which encompasses six additional components to support the meaning of measures, with validity being conceptualized as “a single unitary construct” (Wang and Bai, 2017, p. 932). The unified validity theory places construct validity (i.e., the meaning of measures) at its core and highlights six forms of validity evidence—content, substantive, structural, generalizability, external, and consequential—that can be applied to any measurement of educational or psychological constructs (Messick, 1989, 1995).

Per Messick and other researchers (e.g., Zumbo, 2009; Forer and Zumbo, 2011), these complementary forms of validity evidence cannot function in isolation. Therefore, this study examined two aspects of construct validity to contribute to the effective measurement of SRL strategies and advance the understanding of theoretical functions of SRL for promoting learning achievement. Another aim of this study is to insert some subscales that former studies did not consider and support the suitability of using self-regulation in the EFL context.

### Research questions

The following research questions guided this study:

1. What is the evidence regarding the structural aspects of the construct validity of responses to ESRLQ?
2. What is the evidence regarding the external aspects of the construct validity of responses to ESRLQs?
3. What is the status quo of college students’ use of SRL strategies in Macau and how is the use of these strategies related to English language proficiency?
4. Are there differences between college students’ use of SRL strategies and their English language proficiency across various levels in the English course?

## Materials and methods

### Participants

Volunteer participants were recruited from Macau University of Science and Technology during the first semester of the 2020/2021 academic year. The study was approved by the Research Ethics Committee and carried out in accordance with the institutional requirements. The researchers obtained informed consent from all students before conducting the survey. Meticulous attention was paid to research consent, benefits, privacy, and confidentiality, and participants were informed of their right to withdraw from the study at any time. The sample comprised 598 undergraduate students from 22 classes. Approximately 48% (*n* = 286) were enrolled in a required Level One English course, 42% (*n* = 250) in a Level Two course, and 10% (*n* = 62) in two mixed-level classes for students admitted in or before the 2018/2019 academic year. These students spend 2 h each week in class and approximately two additional hours each week doing homework for the English class. Approximately 43% of the participants were male (*n* = 255) and 57% were female (*n* = 343). Participants’ ages ranged from 16 to 26 (*M* = 18.52, *SD* = 1.33) and their years of English learning ranged from 0 to 23 years (*M* = 11.00, *SD* = 2.75). The participants were from diverse academic faculties, including School of Business, Faculty of Law, Faculty of Chinese Medicine, Faculty of Hospitality and Tourism Management, University International College, and Faculty of Humanities and Arts. The context of leveled and mixed ability groups provided accountable data for further multi-trait comparisons.

The overwhelming majority of Level One/Two participants were in their first year, while the students in the mixed-level classes were juniors or seniors. All students were required to earn required general English education credits in their first academic year, but the university sorted them into different course levels based on their English language test in the *Gaokao* or the *Joint Admission Exam* (both are further elaborated in the following section). As the research was carried out in the autumn term, students with the highest proficiency level were enrolled in Level Two and students at the medium level were in Level One. The two mixed-level classes primarily contained students who had failed either Level One or Level Two in a previous academic term. Lecture periods typically comprised a weekly 3-h session for Level One and Level Two and two 2-h sessions for the mixed-level course.

### Research trajectory

This research progressed through three stages. At the first stage, the conceptual stage was oriented toward questionnaire item generation by exploring the existing literature and consulting with several well-versed practitioners in the field. A pilot study was then conducted in the second stage to determine the framework of ESRLQ. A subsequent interview with 10 first-year students from the 2019/2020 academic year was used to confirm that the items in ESRLQ were appropriate for this population. The third stage involved scrutiny of the psychometric properties of the updated ESRLQ using confirmatory factor analysis.

### Instruments

#### English self-regulated learning questionnaire

A feasible and reliable questionnaire from Wang and Bai (2017) was selected. However, since it was not specifically designed for undergraduate students and that some items were out-of-date, the authors developed and produced a new item pool from which the English self-regulated learning questionnaire (ESRLQ) was finalized. Some items were altered to fit the Macau context after receiving expert reviews and student feedback on the pilot study. For example, two items were least endorsed by participants because both concerned “writing”—something students seldom do now. Accordingly, “Write an outline after reading an English article” was modified to “Rethink its main content after reading an English article,” while “Write learning experience articles or diaries about how I feel about my English learning” became “Keep records for my feeling about my English learning.”

In line with Zimmerman and Risemberg (1997) reconsidered SRL framework, ESRLQ also comprises environmental SRL strategies, behavioral SRL strategies and personal SRL strategies with multiple categories. There are 48 items weaving into 10 dimensions: (a) Self-Evaluation (Items 7, 11, 28, 34, 35, 36); (b) Goal-Setting and Planning (Items 9, 10, 39); (c) Organizing and Transforming (Items 2, 12, 13, 15, 19, 38); (d) Review and Memorization (Items 3, 20, 29, 33); (e) Seeking Social Assistance (Items 6, 14, 16); (f) Persistence (Items 5, 8, 17, 43); (g) Seeking Opportunities (Items 21, 26, 27, 30, 31, 40, 42); (h) Notes Taking (Items 1, 4, 22); (i) Comprehension (Items 18, 23, 24, 25, 32, 37); and (j) Self-Reflection (Items 41, 44, 45, 46, 47, 48). The subscale of self-reflection was first constructed to capture how students interpret and analyze their learning progress, as it may help them self-regulate and solidify what they have learned. A Likert scale of 0–3 was used to measure students’ use of their SRL strategies with 0 standing for “I never use it,” 1 for “seldom use,” 2 for “use it sometimes,” and 3 for “often use.”

#### Final English language exam

The research team received participants’ permission to access their official English final grades at the end of the semester. Participants’ academic achievement was evaluated based on their final reading test score, taken from university records. The grade scale ranges from a minimum of 0 to a maximum of 100, with higher scores indicating greater English language proficiency. For each final, members of the panel of expert or experienced tertiary educators are invited to collaborate in designing the test. They hold meetings to scrutinize the course objectives and make sure the test martial must cover all relevant parts of the course it aims to measure, so as to maintain the norms and the content validity of the exam. The average score of the participants on this exam was 62.56 with a standard deviation of 18.97.

#### English language test in the *Gaokao*

*Gaokao*, a standardized test administered annually in mainland China, is commonly known as the national university entrance exam and also viewed as China’s version of the American SAT and British A-level exams. One of the mandatory *Gaokao* exams is the English language test. The English language test consists of three sections (i.e., reading comprehension, language use, and practical writing) and the full mark of English language test accounts for 150 in most places while only a few provinces have 120 in total score. *Gaokao* English results are important because students with a higher score are assumed to be more proficient in the language, which increases the possibility of being enrolled to a top-tier university. Notwithstanding the fact that some top test-takers in *Gaokao* English exam may be questioned about their practical communication competence with native speakers, universities still accept the score as a basis for direct entry and it also witnesses an uptrend in western institutions to take *Gaokao* result as a measure of academic competence (Farley and Yang, 2019). The validity of *Gaokao* is particularly vital seeing that it plays a decisive role in selecting talents in China. With legitimate concerns on this issue, a number of researchers sought to validate *Gaokao* tests using the conceptual framework coined by long-established experts (e.g., Messick, 1995; Bachman and Palmer, 1996) and constituted proof that *Gaokao* is a kind of norm-referenced test which bears high degree of correlation efficiency between academic performance and *Gaokao* results, and therefore, it has high reliability and validity on average (Tu, 2018; Su, 2021; Zhang, 2021). *Gaokao* English results of the participants in the current study ranged from 20 to 142 with the mean score of 113.44 (*SD* = 21.90).

#### English language test in the joint admission exam

The English Language Test in the joint admission exam (*JAE*) represents a local student’s language proficiency as it is the admission examination jointly organized by four higher education institutions in Macau (i.e., University of Macau, Macau University of Science and Technology, Macau Institute for Tourism Studies, and Macau Polytechnic Institute). Only Chinese, Portuguese, English, and Mathematics are on the *JAE*; other subjects are tested by institutions individually. The *JAE* English language test (*JAE-E*), per the announced syllabus, corresponds to the Common European Framework of Reference (Examination Syllabus and Past Examination Papers, 2021).

The 120-min test is composed of Language Use (40 marks), Reading Comprehension (30 marks), and Essay Writing (30 marks); exam questions are set at a variety of levels, including Elementary (CEFR A2), Pre-Intermediate (CEFR B1), Intermediate (CEFR B2), and Upper-Intermediate/Advanced (CEFR C1). The final mark received by the local student in Macau is generally a weighted sum of their subject marks (the full mark is 1,000). Every year, around ten academic members from the four institutions constitute a committee to jointly organize and administer the exam. To understand the construct and content validation of *JAE-E*, Ho et al. (2021) utilized two instruments (Coh-Metrix and CEFR scales with descriptors) while Kunnan and Yao (2020) drew on teachers’ ratings of task types in *JAE-E*. Their studies specified that on the whole the *JAE-E* was valid with regard to its content. Participants were asked to provide their English *JAE* grade, which ranged from 164 to 807 with a mean of 561.18 (*SD* = 167.34).

As the range of scores differ between the *Gaokao* and *JAE*, all the raw scores were transformed into z-scores and then back-transformed into standardized scores with the same mean (62.56) and standard deviation (18.97) with the final English language Examination. This is reducing the chance of the violation of homogeneity of variance in statistical analysis. The transformed scores keep the rankings of the raw scores.

#### Data analysis

**FIGURE 1:**
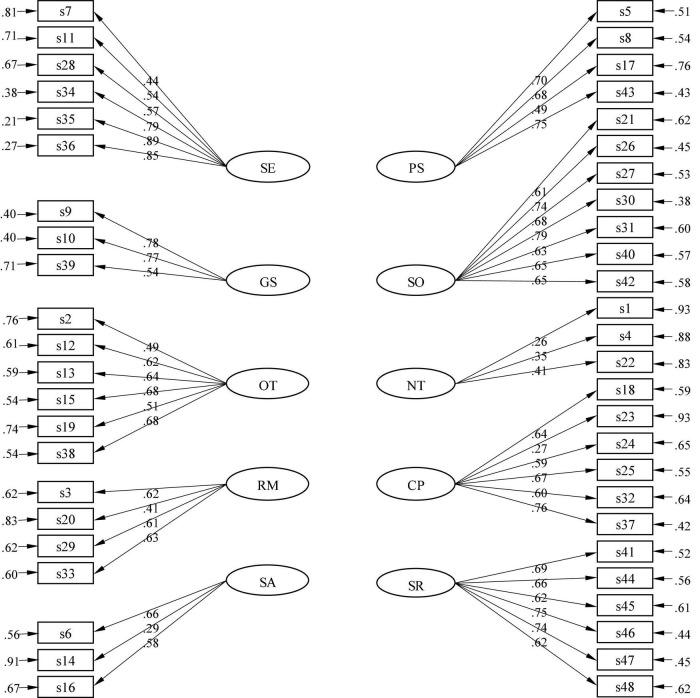
Structure of English self-regulated learning questionnaire. SE, Self-Evaluation; GS, Goal-Setting and Planning; OT, Organizing and Transforming; RM, Review and Memorization; SA, Seeking Social Assistance; PS, Persistence; SO, Seeking Opportunities; NT, Notes Taking; CP, Comprehension; SR, Self-Reflection. All the factors are correlated with each other. The correlations ranged from 0.59 to 0.95 with a mean of 0.76 and standard deviation of 0.10. These correlation coefficients were not presented in figure for the sake of clarity.

The structural aspect of the validity of participants’ responses to the survey was checked with Confirmatory factor analysis (CFA). Instead of using Hu and Bentler’s (1999) two-index strategy in model fit for the goodness of fit indices, a combination of multiple indices was used to judge the model fit: comparative fit index (CFI), incremental fit index (IFI), standardized root mean square residual (SRMR), root mean square error of approximation (RMSEA), and the 90% confidence intervals of RMSEA. This is because some research studies have questioned the validity of the two-index strategy suggested by Hu and Bentler (1999) in model fit assessment (Marsh et al., 2004; Fan and Sivo, 2005). The suggestions to add paths from observable to latent variables were not followed either to avoid mechanically fitting the model without theoretical justifications (MacCallum et al., 1992). Error covariances between observable variables within each latent construct were not added (Figure 1).

Pearson correlation coefficients were used to check the external aspects of the construct validity by correlating the use of SRL strategies measured by ESRLQ with student performance on two English examinations: *Gaokao*/*JAE* English test score for comprehensive English language proficiency and the final English exam score measured at the end of the semester for English language reading competence. Multivariate analysis of covariance (MANCOVA) was employed to compare the SRL strategy use as well as the comprehensive English language proficiency and English language reading competence between the three cohorts: Level 1, Level 2, and Mixed Levels. The number of years studying English was used as a covariant. Effect size (partial eta squared) was reported as small (0.01), medium (0.06), or large (0.14) according to Cohen (1988).

## Results

Responses to the survey items were found to be consistent. Internal consistency measured by Cronbach’s value ranged from 0.82 to 0.96 for each subscale. These results support the reliability of responses to ESRLQ. Results from CFA showed that the responses to survey items fall into the expected structure: 10 factors of ESRLQ. The data fit the model well. The goodness-of-fit indices were all satisfactory: CFI > 0.90, SRMR < 0.07, and RMSEA < 0.08 (Table 1). Moreover, the factor loadings of items to each factor of ESRLQ were all statistically significant and were mostly above 0.50 (Figure 1). As a result, findings of this study support the structural aspect of the construct validity of ESRLQ. Statistically significant relationships were also reported between the use of self-regulated learning strategies and student’s comprehensive English language proficiency, *r* = 0.47, *p* < 0.001, as well as student’s English language reading competence, *r* = 0.46, *p* < 0.001, both with a large effect size (Table 2). Table 2 also reports the correlations between the 10 factors of ESRLQ and their associations with the total SRL use, comprehensive English language proficiency, and English language reading competence, providing evidence for the external aspect of the construct validity of ESRLQ.

**TABLE 1:** Fit indices for the measurement model.

_χ^2^_	*df*	CFI	IFI	SRMR	RMSEA	LL	UL
3,625.09	1,035	0.97	0.97	0.067	0.064	0.062	0.066

CFI, comparative fit index; IFI, incremental fit index; SRMR, standardized root mean square residual; RMSEA, room mean square error of approximation. LL refers to the lower limit and UL refers to the upper limit of the 90% confidence interval of RMSEA.

**TABLE 2:** Correlations between factors of SRL strategy use, comprehensive English proficiency, and English reading competence.

	GS	OT	RM	SA	PS	SO	NT	CP	SR	Total	CEP	ERC
SE	0.62	0.64	0.53	0.46	0.61	0.71	0.49	0.56	0.70	0.85	0.37	0.39
GS	–	0.63	0.54	0.49	0.59	0.59	0.57	0.46	0.67	0.78	0.29	0.34
OT		–	0.56	0.45	0.62	0.59	0.51	0.66	0.69	0.84	0.40	0.39
RM			–	0.44	0.51	0.42	0.48	0.47	0.59	0.70	0.27	0.24
SA				–	0.46	0.43	0.40	0.40	0.54	0.62	0.30	0.26
PS					–	0.68	0.41	0.58	0.64	0.80	0.49	0.47
SO						–	0.44	0.51	0.62	0.82	0.41	0.40
NT							–	0.31	0.55	0.63	0.19	0.20
CP								–	0.58	0.73	0.47	0.41
SR									–	0.86	0.36	0.35
Total										–	0.47	0.46
CEP											–	0.62
ERC												–

SE, Self-Evaluation; GS, Goal-Setting and Planning; OT, Organizing and Transforming; RM, Review and Memorization; SA, Seeking Social Assistance; PS, Persistence; SO, Seeking Opportunities; NT, Notes Taking; CP, Comprehension; SR, Self-Reflection; CEP, Comprehensive English language proficiency; ERC, English language Reading Competence. All correlations are significant at *p* < 0.01.

Descriptive statistics of the use of SRL strategies by students in the three cohorts are in Table 3. According to the descriptive statistics, the participants used some SRL strategies as the mean scores for all cohorts ranged between 1 (I seldom use it) and 3 (I often use it). For example, participants reported the use of the following strategies the most: comprehension, seeking social assistance, and persistence. The positive findings are that the use of comprehension and persistence strategies was also strongly associated with the participant’s comprehensive English language proficiency and English language reading competence (*r* ranged from 0.41 to 0.49). However, participants reported the least use of seeking opportunities to practice the English language although the use of this strategy was strongly associated with the comprehensive English language proficiency (*r* = 0.40) and English language reading competence (*r* = 0.41).

**TABLE 3:** Means and standard deviations for SRL strategy use, comprehensive English proficiency, and English reading competence.

	Cohorts			
	
	Level 1 (*n* = 286)	Level 2 (*n* = 250)	Mixed (*n* = 62)	All (*n* = 598)
SE	1.74 (0.63)	2.01 (0.58)	1.42 (0.64)	1.82 (0.64)
GS	1.84 (0.64)	2.10 (0.63)	1.49 (0.61)	1.92 (0.66)
OT	2.00 (0.56)	2.26 (0.47)	1.60 (0.61)	2.07 (0.56)
RM	2.07 (0.54)	2.23 (0.58)	1.72 (0.64)	2.10 (0.59)
SA	2.24 (0.53)	2.35 (0.47)	1.91 (0.57)	2.26 (0.53)
PS	2.12 (0.57)	2.48 (0.43)	1.71 (0.59)	2.23 (0.57)
SO	1.56 (0.61)	1.92 (0.56)	1.30 (0.60)	1.68 (0.62)
NT	1.82 (0.47)	1.93 (0.51)	1.54 (0.52)	1.84 (0.50)
CP	2.33 (0.48)	2.60 (0.35)	1.85 (0.61)	2.40 (0.49)
SR	2.03 (0.59)	2.26 (0.55)	1.62 (0.57)	2.09 (0.60)
Total	1.97 (0.44)	2.21 (0.37)	1.59 (0.50)	2.05 (0.45)
CEP	57.84 (16.36)	74.60 (5.51)	28.77 (21.21)	62.76 (18.66)
ERC	61.33 (20.36)	68.74 (12.98)	37.18 (14.43)	62.56 (18.97)

SE, Self-Evaluation; GS, Goal-Setting and Planning; OT, Organizing and Transforming; RM, Review and Memorization; SA, Seeking Social Assistance; PS, Persistence; SO, Seeking Opportunities; NT, Notes Taking; CP, Comprehension; SR, Self-Reflection; CEP, Comprehensive English language proficiency; ERC, English language Reading Competence. Numbers in parentheses are standard deviations.

The linear relationship between the number of years studying English and the English language proficiency score was statistically significant, *r* = 0.30, *p* < 0.01. As a result, the assumption to use MANCOVA to compare the SRL strategy use between the students from the three cohorts: Level 1, Level 2, and Mixed Levels was met. Statistically significant differences were found in the linear combination of all three dependent variables, Wilks’ lambda = 0.50, *F*(6, 1110) = 76.36, *p* < 0.001, partial η^2^ = 0.29 (large effect). Tests of between-subjects effects suggested statistically significant differences in the use of SRL strategies, *F*(2, 557) = 47.62, *p* < 0.001, partial η^2^ = 0.15 (large effect); comprehensive English language proficiency, *F*(2, 557) = 258.92, *p* < 0.001, partial η^2^ = 0.48 (large effect); and English language reading competence, *F*(2, 557) = 64.44, *p* < 0.001, partial η^2^ = 0.19 (large effect). *Post hoc* multiple comparisons noted that students in Level Two (advanced level) scored more than their peers in Level One (medium level) and that students in Level One scored more than their counterparts in Mixed Level (those who failed in Level One or Level Two) in all three dependent variables, namely, use of SRL strategies, comprehensive English language proficiency, and English language reading competence.

## Discussion

SRL theory has been promoted to examine and foster students’ functioning in academic contexts (Zimmerman, 1990; Schunk and Zimmerman, 2012; Theobald, 2021). The current study integrated the measurement of SRL and the associations between the use of SRL strategies and academic achievements. Three cohorts of participants (advanced-level, medium-level, and mixed-level) were compared for the use of SRL strategies and their performance on English language proficiency and English language reading comprehension tests. This study provided evidence for the structural and external aspect of construct validity of the new instrument.

Future researchers may adopt this instrument to measure Chinese college students’ use of SRL strategies, especially in the region of Macau (and maybe Hong Kong) where student characteristics differ from those in mainland China. Students in Macau and Hong Kong are similar to students in mainland China in that they are all Chinese in ethnicity but different from students in mainland China in that they inherit quite a lot western culture due to the history. Another difference is that English is used much more often in both school settings and social context in Macau and Hong Kong.

Also noteworthy was that in our study, advanced-level students reported significantly more frequent use of self-regulated learning strategies and scored higher than their peers in comprehensive English language proficiency test and English language reading competence test. The positive relationship between the use of SRL strategies and academic achievement reported in this study echoed previous research with learning strategies in the field of teaching/learning English as a foreign/second language (e.g., Bai et al., 2019, Chen et al., 2020).

Empirical evidence emerged in our research also supports the social cognitive theory (Bandura, 1986) and previous research on self-regulation and language learning strategies (i.e., Oxford, 1990, 1996, 2011, 2015; Zimmerman, 2000; Bai and Wang, 2020; Sun et al., 2022). As a result, this study contributes to the theory of self-regulation. Specifically, the structure of the ESRLQ reflected the reciprocity within social cognitive theorizing, involving a triadic process: self-observation, self-judgment, and self-reaction (Kanfer and Gaelick, 1986; Schunk, 1989; Zimmerman, 1989).

## Conclusion and implications

The findings of our study provided evidence for the reliability and validity of student responses to the survey to measure the use of SRL strategies. First, the internal structure of the instrument to measure the use of SRL strategies remained consistent with the previous version from which it was adapted (Wang and Bai, 2017). The external aspects of the construct validity were checked with correlations between the use of SRL strategies and students’ performance on both standardized tests and final exams in the English reading course, with statistically significant relationships to support our expectations. These results are in line with theoretical expectations as well as previous studies in terms of both direction and magnitude (e.g., Broadbent and Poon, 2015; Akamatsu et al., 2019; Sun and Wang, 2020; Bai and Wang, 2020).

Descriptive statistics of the use of SRL strategies suggest that college students in Macau use some SRL strategies but not very often. Strategies under the category of Seeking Opportunities (e.g., read English journals/newspapers/magazines on my initiative and watch English programs on my initiative) were least used. One possible reason is that these strategies are more related to students’ intrinsic motivation rather than teachers’ requirement in English reading courses. While societal norms and cultural values in Macau may have an impact on students’ learning intention and behavior—in absence of a positive sense of wellbeing, students tend to follow teachers’ request and are not often trained to take initiatives for their learning (Cheong, 2022). This implies a need for them to become more self-aware, self-sustaining and self-efficacious. As noted by Broadbent and Poon (2015), learning is more effective if the participant knows how to make and adjust plans to attain academic success. With proper use of SRL strategies, learners are more likely to master the course content and achieve expected learning outcomes (e.g., Chang, 2005; Moseki and Schulze, 2010).

English language instructors may need to adopt SRL strategy development approach to help students gain more SRL strategies. A recent meta-analysis of 22 primary studies has provided a strong and positive link between the use of SRL strategies and language learning outcomes (Sun et al., 2022). In this study, we have advanced the SRL literature by introducing a new instrument to measure college students’ use of SRL strategies. The findings from this study provided more evidence to support the SRL theory in the context of learning EFL.

This study also has practical implications for teachers of ESL/EFL. The findings of this study echoed previous empirical studies and provided evidence to support the strong and positive associations between the use of SRL strategies and English language proficiency. Teachers of English are encouraged to participate in professional development workshops and to learn how to explicitly and systematically incorporate the use of SRL strategies in their classroom instruction so that EFL/ESL learners can benefit more from the English language course. With the help of their teachers, ESL/EFL learners may become adaptive learners with self-regulative process who know how to set goals, manage resources, and monitor their progress, positive learners who may sustain interest and maintain confidence, and proactive learners who know how to select the most appropriate strategies for themselves (Zimmerman, 2002, 2008). This study is significant in that it calls for teachers’ attention to help students develop effective learning strategies while learning ESL/EFL, which will likely improve the efficiency and effectiveness of students’ study at college.

### Limitations

Future researchers are encouraged to have a closer examination of the differences between deep and surface cognitive strategies, which was not examined within the current study. Another limitation of this study lies in the small target population. Although the students in Macau were rarely studied in the past, they are a special Chinese group because they do not have to take *Gaokao* like other Chinese students do. The JAE test was specially tailored for them. Therefore, test equivalence between *Gaokao* and JAE is another direction for psychometricians in the future. Finally, readers should be cautious when interpreting the results and generalizing the findings to their own population (e.g., other formerly colonized regions).

## Data availability statement

The raw data supporting the conclusions of this article will be made available by the authors, without undue reservation.

## Ethics statement

The studies involving human participants were reviewed and approved by Macau University of Science and Technology. The participants provided their written informed consent to participate in this study.

## Author contributions

XD: data collection and manuscript writing. CW: conceptualization, data analysis, and reviewing and editing. JX: reviewing and editing. All authors contributed to the article and approved the submitted version.

## Appendix

Appendix: English self-regulated learning questionnaire (ESRLQ).

Part One (Personal information)

Student ID: _______________________ Class code: ___________ Gender: ________ Age: _____

Faculty: ______________ Grade: ________ The number of years you have learned English: _____

Your English test score in the *Gaokao* (if applicable): ____________

Your English test score in the *JAE* (if applicable): ____________

Willing to be interviewed: Yes / No

Part Two (SRL strategies)

Please choose answers from the following study methods according to your actual situation. Please notice that this is not a test, so there are no right or wrong answers. Not all the methods listed here are good methods, and everyone has his/her own methods. We intend to know which methods are those you actually use and the frequency of using them. Thanks for your participation!

0 = I never use it. 1 = I seldom use it. 2 = I use it sometimes. 3 = I often use it.

**Table:**

The Statement of Self-Regulated Learning Strategies				
1. Write down the mistakes I often make in the process of studying English.	0	1	2	3
2. Make an outline or mind-map before writing English essays.	0	1	2	3
3. Review English texts I have learned.	0	1	2	3
4. Take notes in English classes.	0	1	2	3
5. Keep reading when I encounter difficulties in English reading.	0	1	2	3
6. Consult teachers when I encounter difficulties in the process of studying English.	0	1	2	3
7. Check my English homework before turning them in.	0	1	2	3
8. Read an English article several times if I don’t understand it at the first time.	0	1	2	3
9. Make a study plan in the process of studying English.	0	1	2	3
10. I have definite (clear) goals to study English.	0	1	2	3
11. Use the answers in English course materials or web pages to check my acquisition of what I have learned.	0	1	2	3
12. Rethink its main content after reading an English article.	0	1	2	3
13. Summarize the main idea of each paragraph when reading.	0	1	2	3
14. Search relevant information online when I have difficulties in studying English.	0	1	2	3
15. Summarize the theme of an English article when I read it.	0	1	2	3
16. Ask classmates/friends when I have questions in my English study.	0	1	2	3
17. Practice my pronunciation through listening to an English program several times.	0	1	2	3
18. Guess the meaning of new words by considering their contexts.	0	1	2	3
19. Classify news words in order to memorize them.	0	1	2	3
20. Write new words many times in order to memorize the spellings.	0	1	2	3
21. Use sentence patterns just learned to make new sentences for practice.	0	1	2	3
22. Keep records for my feeling about my English learning.	0	1	2	3
23. When I come across a new English word in reading, I skip it and move on if it does not hinder my comprehension.	0	1	2	3
24. In English reading or conversations, I will predict what is to come according to what I have already known.	0	1	2	3
25. When I read an English article, I imagine the scene described in the article in order to memorize what I have read.	0	1	2	3
26. Read English journals/newspapers/magazines on my initiative.	0	1	2	3
27. Have conversations in English with teachers, classmates or friends (especially foreign friends) to practice my English.	0	1	2	3
28. I ask myself questions to check whether I understand what I have learned in the English course.	0	1	2	3
29. Read new words repeatedly in order to memorize them.	0	1	2	3
30. I look for opportunities to talk in English.	0	1	2	3
31. Watch English programs on my initiative.	0	1	2	3
32. Memorize meanings of words by using prefixes and suffixes.	0	1	2	3
33. Review my notes of English class before examinations.	0	1	2	3
34. I conduct self-assessments on my English communicative ability.	0	1	2	3
35. I conduct self-assessments on my English writing ability.	0	1	2	3
36. I conduct self-assessments on my English reading ability.	0	1	2	3
37. Use my background knowledge to comprehend English articles.	0	1	2	3
38. Make sure that the content of each paragraph supports its topic sentence in English writing.	0	1	2	3
39. When learning a new English course, I pay close attention to the course syllabus, content and assessment forms.	0	1	2	3
40. Seek theme-related materials for my further reading.	0	1	2	3
41. Make summaries after reading an English chapter or taking the chapter quiz.	0	1	2	3
42. I attempt to think in English when I use it.	0	1	2	3
43. Persist in my English study even though I encounter difficulties.	0	1	2	3
44. I endeavor to find out how to better my English learning.	0	1	2	3
45. I talk to someone else about how I feel about learning English.	0	1	2	3
46. I reflect on my learning performance through the evaluation of English assignments or exams.	0	1	2	3
47. I use my teacher’s feedback to adjust my English learning in the following phase.	0	1	2	3
48. I use my classmates’/friends’ feedback to adjust my English learning in the following phase.	0	1	2	3
